# Low-Cost Impact Detection and Location for Automated Inspections of 3D Metallic Based Structures

**DOI:** 10.3390/s150612651

**Published:** 2015-05-28

**Authors:** Carlos Morón, Marina P. Portilla, José A. Somolinos, Rafael Morales

**Affiliations:** 1 Sensors and Actuators Group, Universidad Politécnica de Madrid, Juan de Herrera 6, Madrid 28040, Spain; E-Mail: carlos.moron@upm.es; 2 GITERM. ETSI. Navales, Universidad Politécnica de Madrid, Arco de la Victoria 4, Madrid 28040, Spain; E-Mails: marinap.portilla@upm.es (M.P.P.); joseandres.somolinos@upm.es (J.A.S.); 3 E.T.S.I. Industriales, Universidad de Castilla-La Mancha, Albacete 02071, Spain

**Keywords:** collision detection, metallic structures, acoustic wave propagation, robotized inspection

## Abstract

This paper describes a new low-cost means to detect and locate mechanical impacts (collisions) on a 3D metal-based structure. We employ the simple and reasonably hypothesis that the use of a homogeneous material will allow certain details of the impact to be automatically determined by measuring the time delays of acoustic wave propagation throughout the 3D structure. The location of strategic piezoelectric sensors on the structure and an electronic-computerized system has allowed us to determine the instant and position at which the impact is produced. The proposed automatic system allows us to fully integrate impact point detection and the task of inspecting the point or zone at which this impact occurs. What is more, the proposed method can be easily integrated into a robot-based inspection system capable of moving over 3D metallic structures, thus avoiding (or minimizing) the need for direct human intervention. Experimental results are provided to show the effectiveness of the proposed approach.

## 1. Introduction

During the last few years various climbing robots have been developed for different inspection purpose applications. Briones *et al.* [1] developed a climbing robot for inspection in nuclear plants; Minor *et al.* [2,3] designed miniaturized climbing robots with special under-actuated kinematics. Other authors have developed a family of multifunctional autonomous self-supported climbing robots capable of traveling in complex metal-based environments [4,5,6]. Furthermore, metal-based structures are very common in the construction sector, and as an example of civil infrastructure, it is estimated that there are more than 42,000 steel bridges in the EU. The use of an autonomous climbing robot to inspect this type of structure avoids or reduces the need for a direct human presence, which is an important advantage from the viewpoint of safety and human resources.

Inspection techniques are very important for improving the safety and reliability of aged structures. Different methods can be found in the scientific literature such as infrared temperature measurement [7], ultrasonic C-scans [8] or X-rays [9]. Other recent techniques showing high effectiveness in the evaluation of damaged structures are based on Lamb wave visualization techniques [10,11]. The inspection tasks on metal-based structures can be organized into two groups: the first group covers the periodical inspection of bolted or welded unions, inspection of the painting of the structure or inspections to detect corrosion or damage to the structure, among others. The second group is related to the non-scheduled inspection that is required when a particular unplanned event has occurred and it is necessary to evaluate its effects on the structure. One of the most common events that could require inspection is when an impact or collision occurs. For the first type of periodical inspection, the robot uses a geometrical description of the structure (CAD), programmed target points and robot path planning abilities to cover the whole or part of the structure. For the second type of unexpected inspection, only the zone of interest has to be reached and inspected. In these cases, the first step consists of detecting and locating the point at which this impact has occurred, after which a climbing robot will access this zone. The robot can now be directed by means of teleoperation or by autonomous path planning in the neighborhood of the desired point. However, if it is not possible to locate the point of the impact, there is no path planning input data for the navigation system, and the robot cannot be provided with a target point.

This paper is focused on the development of a new automatic means to detect and locate mechanical impacts (collisions) on 3D metal-based structures, thus helping to improve the possible tasks related to the robotized inspection of the point or zone of impact and avoiding (or reducing) direct human intervention on the structure. Various automatic methods to detect impacts have been developed in different fields: Shin *et al.* [12] developed methods for accurately measuring the arrival time delay between two sensors attached to a duct line system. In [13] a method of identifying the impact force for composite structures based on the relation between force histories and strain responses is presented. Atobe *et al.* [14] developed in recently reported studies a method for monitoring the impact damage in FRP pressure vessels based on force identification. García *et al.* [15,16] detected collisions on the tip of a flexible robot by processing gauge signals. Several methods are currently used to detect the moment of impact, but they are unable to obtain its position. Examples of this kind of applications are car airbag switches [17,18] or particle impact detectors [19]. The proposed method for locating and detecting an impact on a metallic bar [20,21,22] and flat surfaces [23] based on the propagation of acoustic signals on structures [24] is generalized in this paper in order to detect and locate an impact or collision on a 3D general structure.

The remainder of the paper is structured as follows: Section 2 describes the method used to detect and locate impacts on a single bar and on a 3D structure. The laboratory experimental setup used to carry out the experiments is then explained in Section 3. More specifically, a description of the electronic circuit used to measure time delays and the geometrical description of the structure and sensors are provided. Section 4 presents the experimental results obtained with the proposed algorithm. Finally, Section 5 is devoted to the conclusions and proposals for future work.

## 2. Detection and Location of an Impact

This section provides a description of the method used to detect and locate an impact on a single bar. A generalization of this method is then applied to a general 3D structure.

### 2.1. Location of an Impact on a Single Bar

**Figure 1:**
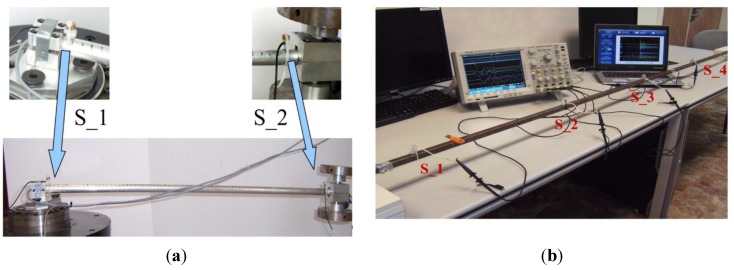
Experimental setup for an impact on a single bar with: (**a**) two piezoelectric sensors; (**b**) four piezoelectric sensors.

A single bar could be considered as the simplest structure. When an impact is produced on this bar, two acoustic waves travel from the impact point to both ends of the bar. Under the hypothesis that the bar is constructed of a homogeneous and not overly acoustic attenuating material, the time that the waves take to reach each end is proportional to the distance from the impact point to both ends. The method used to determine the instant at which the impact has been produced consists of detecting the acoustic wave generated as soon as possible using piezoelectric sensors. If two piezoelectric sensors are positioned at both ends of the bar (see Figure 1), the propagation times of the acoustic waves can be calculated using the following expressions:

(1) $$t_{1}=\frac{x}{C_{m}}\;;\;\;\;\;\;\;\;\;\;t_{2}=\frac{L-x}{C_{m}}$$

where L is the length of the bar, x is the distance between the impact point and the sensor S_1, L−x is the distance between the impact point and the sensor S_2, $C_{m}$ is the sound propagation speed, and $t_{1}$ and $t_{2}$ are the times of propagation.

The difference between times $t_{1}$ and $t_{2}$ is denoted as $∆t_{12}=t_{2}-t_{1}$ or $∆t_{21}=t_{1}-t_{2}$ and it can be measured directly, rather than measuring $t_{1}$ or $t_{2}$:

(2) $$∆t_{12}=t_{2}-t_{1}=\frac{L-2x}{C_{m}}\;;\;\;\;\;\;\;\;\;\;∆t_{21}=t_{1}-t_{2}=\frac{2x-L}{C_{m}}$$

If L and $C_{m}$ are known, then the position of x is determined by using the following pair of equations:

(3) $$x=\frac{L-∆t_{12}C_{m}}{2}\;;\;\;\;\;\;\;\;\;\;x=\frac{L+∆t_{21}C_{m}}{2}$$

Equation (3) has three possibilities as a function of the relative position of x (impact) with regard to the center of the bar:

(4) $$\{\begin{array}{c} ∆t_{12}>0\;\;\;\;if\;\;\;\;\;\;x<\frac{L}{2}\\ ∆t_{12}=0\;\;\;\;if\;\;\;\;\;\;x=\frac{L}{2}\\ ∆t_{12}<0\;\;\;\;if\;\;\;\;\;\;x>\frac{L}{2} \end{array}\;;\;\;\;\;\;\;\;\;\;\{\begin{array}{c} ∆t_{21}>0\;\;\;\;if\;\;\;\;\;\;L-x<\frac{L}{2}\\ ∆t_{21}=0\;\;\;\;if\;\;\;\;\;\;L-x=\frac{L}{2}\\ ∆t_{21}<0\;\;\;\;if\;\;\;\;\;\;L-x>\frac{L}{2} \end{array}$$

If both sensors detect the wave at the same time, the impact position is easily determined by any of the Equations (3) as $x=\frac{L}{2}$*.*

The absolute instant of impact is meanwhile approximately obtained from the first sensor which detects the wave. If the exact instant of impact is required, it can be obtained from the estimated position of the impact $x_{estimated}$ using Equations (1) and (3) as follows:

(5) $$t_{impact}=t_{1}-\frac{x_{estimated}}{C_{m}}\;;\;\;\;\;\;\;\;\;\;t_{impact}=t_{2}-\frac{L-x_{estimated}}{C_{m}}$$

In order to verify the equations which have been obtained from the above analysis, the situation in which there are four sensors was considered (see Figure 1b). The difference between this case and the former is that two sensors are located at the bar ends while another two are arranged in the central zone so that the bar is divided into three equal areas. Note that only the two sensors closest to both sides of the place of impact are taken into account (for details see [25]). In short, the problem as regards the case of two sensors is reduced.

**Figure 2:**
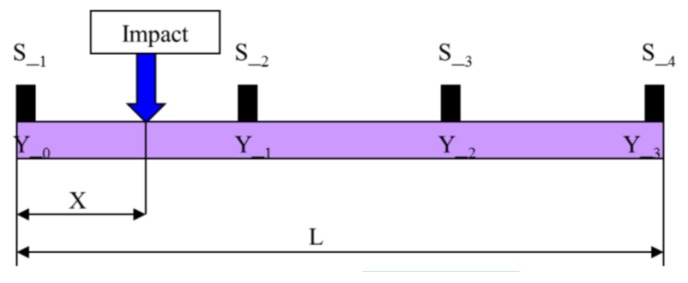
Mathematical modeling for an impact on a single bar with four piezoelectric sensors.

As can be easily deduced, we have three different possibilities, *i.e.*, when the impact occurs between sensors 1-2, 2-3 or 3-4. The distance L is divided into K−1 equidistant lengths (in our case K=4) where the distance to the first sensor S_1 is denoted by $Y_{n}$. For example, if we consider that the impact occurs between sensors 1 and 2 (see Figure 2), this yields:

(6) $$Y_{n}=n\frac{L}{K-1}\;\;\text{ with }K=4\;\text{ and}\;0\;\leq n\;\leq 3$$

so:

(7) $$\begin{array}{c} t_{1}=\frac{X}{C_{m}}\;\\ t_{2}=\frac{1}{C_{m}}\left[\frac{L}{K-1}-x\right] \end{array}\}\;\Rightarrow \;{\text{Δt}}_{12}=t_{1}-t_{2}=\frac{1}{C_{m}}\left[2x-Y_{1}\right]\;\;\to \;\;\;x=\frac{1}{2}\left[{\text{Δt}}_{12}{\text{C}}_{m}+Y_{1}\right]$$

**Figure 3:**
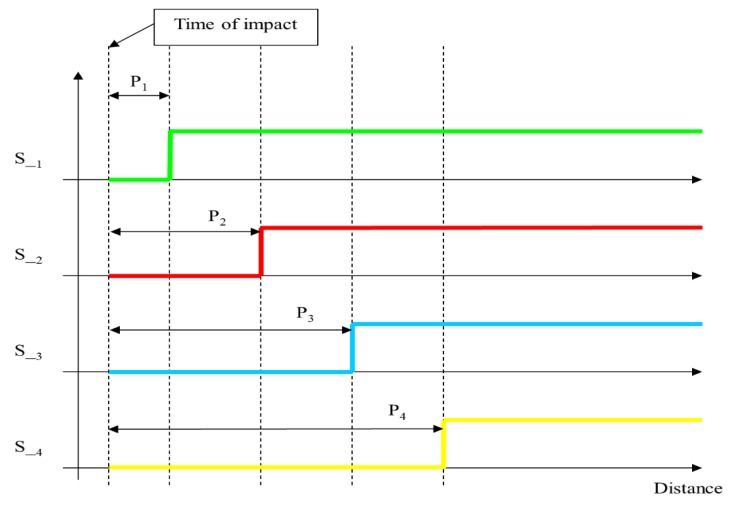
Time of impact on a single bar between sensors 1 and 2.

Figure 3 shows the sequence of signals at the different sensors, with the respective times obtained for the position and speed of propagation. Assuming that $P_{1}$, $P_{2}$, $P_{3}$ and $P_{4}$ are respectively the distances from the sensors 1, 2, 3, 4 to the impact point, these phases are:

(8) $$\begin{array}{c} \text{Δ}t_{21}=\frac{P_{2}-P_{1}}{C_{m}}\\ \text{Δ}t_{32}=\frac{P_{3}-P_{2}}{C_{m}}\\ \text{Δ}t_{43}=\frac{P_{4}-P_{3}}{C_{m}} \end{array}$$

If the impact is between sensors 2-3 or 3-4, the results are similar. In short, if we start by assuming propagation at a constant speed on the bar, and if the impact occurs between two correlative sensors, the time differences that are expected to be detected by the other sensors are given by the following equation:

(9) $$\text{Δ}t_{ij}=\frac{P_{xi}-P_{xj}}{C_{m}}$$

Note that if this phase is not maintained between the correlated pairs of sensors, it must be taken into account that an attenuation occurs in those furthest away from the impact position. It is for this reason that in this study we have considered using only two sensors located on both sides of the impact position.

### 2.2. Locating an Impact on a 3D Structure

Let us consider a general 3D structure given by its geometrical CAD model. If a group of n piezoelectric sensors is located strategically along the structure, and taking into account that the acoustic wave from a point to any of the sensors will use the path of minimum distance, the structure can be modeled as the location of these *n* sensors (S_1, S_2, …, S_n) and a matrix D of minimum distances between sensors (1-dimensional distances):

(10) $$D=\left[\begin{array}{cccc} d_{11} & d_{12} & \cdots & d_{1n}\\ d_{21} & d_{22} & \cdots & d_{2n}\\ \vdots & \vdots & \ddots & \vdots \\ d_{n1} & d_{n2} & \cdots & d_{nn} \end{array}\right]$$

One of the structural properties of matrix D (shown in Equation (10)) is that D is symmetrical ($D=D^{T}$), that is to say, $d_{ij}=d_{ji}$, ∀ 1≤i,j≤n with i≠j and $d_{ii}=0$, ∀ 1≤i≤n. Elements $d_{ij}$ denote the minimum distances between sensor S_i and S_j. For n sensors, only $\frac{{\left(n-1\right)}^{2}}{2}$ rather than $n^{2}$ values are necessary.

When an impact is produced on the 3D structure, the acoustic waves travel along it from this point to each sensor. These detection times are denoted as:

(11) $$T=\left[\begin{array}{cccc} t_{1} & t_{2} & \cdots & t_{n} \end{array}\right]$$

where $t_{i}$, ∀ 1≤i≤n denotes the time of propagation from the impact point to the sensor S_i. As in the previous case of a single bar, these times cannot be measured directly. Only differences between times can be obtained. If sensor S_i is the first to detect the wave, we can obtain the differences between times from this $i^{th}$-sensor. Equation (10) is then adapted to:

(12) $$\Delta t_{i\_s}=\left[\begin{array}{ccccc} 0 & \text{Δ}t_{i\left(2\_s\right)} & \text{Δ}t_{i\left(3\_s\right)} & \cdots & \text{Δ}t_{i\left(n\_s\right)} \end{array}\right]$$

$\text{Δ}t_{i\left(j\_s\right)}$, ∀ 2≤j≤n now denotes the differences in time of propagation between sensors S_i and S_(j_s). The distances between sensors S_i and S_(j_s) form the $i^{th}$ row of matrix D, and this is denoted as $D_{i}$:

(13) $$D_{i}=\left[\begin{array}{ccccccc} d_{i1} & \cdots & d_{i\left(i-1\right)} & 0 & d_{i\left(i+1\right)} & \cdots & d_{in} \end{array}\right]$$

**Figure 4:**
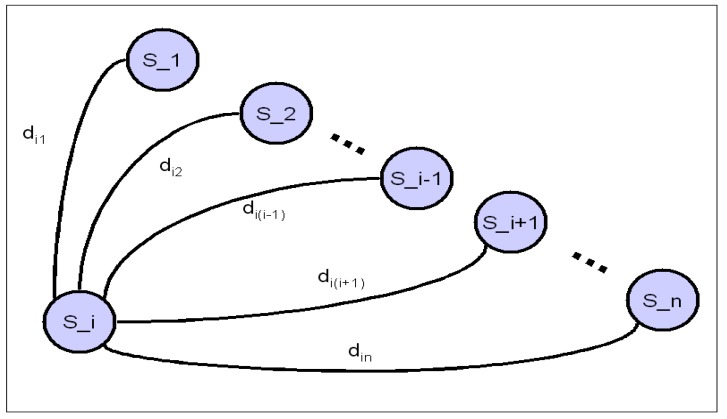
Distances from S_i to each sensor on the structure.

Figure 4 represents the reduced topological representation of the structure according to Equation (11). All the distances are considered to be single bars.

A similar equation to (3) is now used to obtain the relative position $x_{i\left(j\_st\right)}$ with regard to S_i and is measured along the path between S_i and S_(j_s). It can be observed that the equation below is a generalization of Equation (3) for any pair of sensors:

(14) $$x_{i\left(j\_s\right)}=\frac{d_{i\left(j\_s\right)}-\text{Δ}t_{i\left(j\_s\right)}C_{m}}{2}$$

for every j, ∀ 2≤j≤n.

From Equation (11), n−1 solutions can be obtained, and some of these solutions allow us to determine the position of impact. Owing to the attenuation, loss of linearity of propagation, *etc.*, it should be clear that for larger values of $\text{Δ}t_{i\left(j\_s\right)}$—or the index j, the precision of detection of the wave deteriorates. It is not, however, necessary to obtain the n−1 solutions, since the first solution alone allows us to determine the position of impact. If more than one solution is obtained, they can be analyzed in order to check the goodness of the solutions, thus avoiding mistakes, *etc.*

The instant of impact is approximately obtained from the first sensor, which detects the wave. If the exact time of impact is required, it can be estimated using Equations (9) and (13):

(15) $$t_{impact}=t_{i}-\frac{x_{i{\left(j\_st\right)}_{estimated}}}{C_{m}}$$

## 3. Experimental Setup

**Figure 5:**
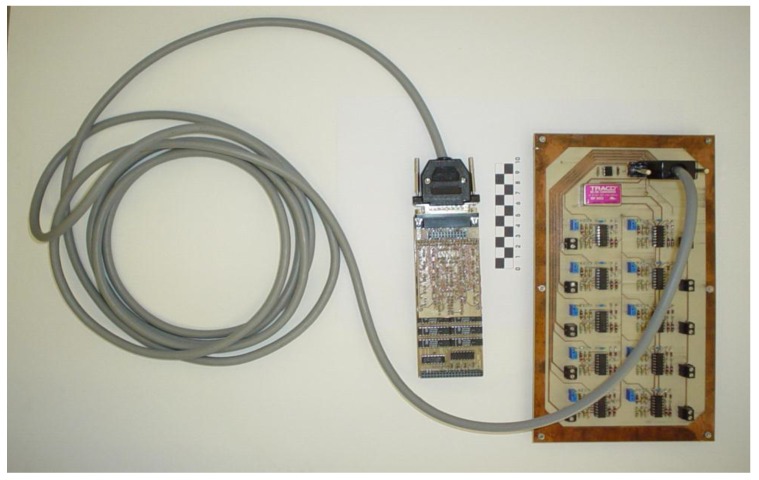
Electronic circuit for time delays measurements.

A special electronic circuit for measuring time delays was used in experiments (Figure 5). This circuit basically consists of an external board which is in charge of converting the original voltage signals obtained from piezoelectric sensors into edge signals. Each of the ten channels was designed with the following four stages (all of them implemented with operational amplifiers): (i) a voltage follower; (ii) a full-wave rectifier precision; (iii) a signal level adaptation and an inverting stage and (iv) a Schmitt Trigger stage with an trimmer-based adjustable threshold and an external reset terminal. Then, the edge signals are sent through a transmission multiline from the analog external board to the opto-isolation and digital board. Special attention was paid to mass, screen or grid connections. A brief description of the hardware used can be found in [22] and a very detailed description of it can be found at [26].

**Figure 6:**
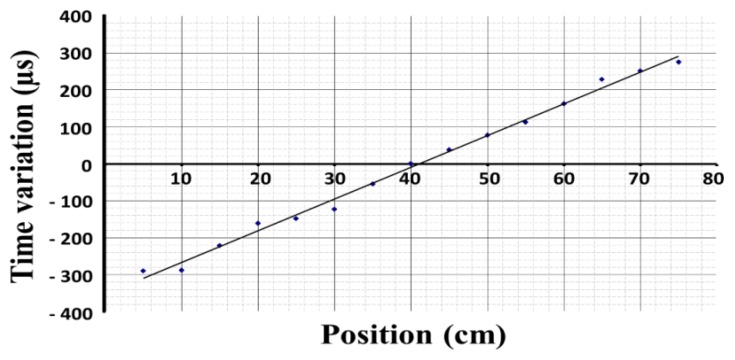
Calibration curve for an 80 cm bar.

The opto-isolation and digital stages have been implemented as a standard *m*-module board in according with the VITA *m*-module specification. The first prototype was built using discrete components while the current digital stage of the prototype was implemented on a Xilinx FPGA. For the experimental measurements we have used a detection level of 2 V and a clock frequency of 1 MHz. A lower level of detection implies that the system is highly sensitive, whereas a high clock frequency provides a high resolution. We have thus obtained the calibration curve for the 0.8 m bar shown in Figure 6.

**Figure 7:**
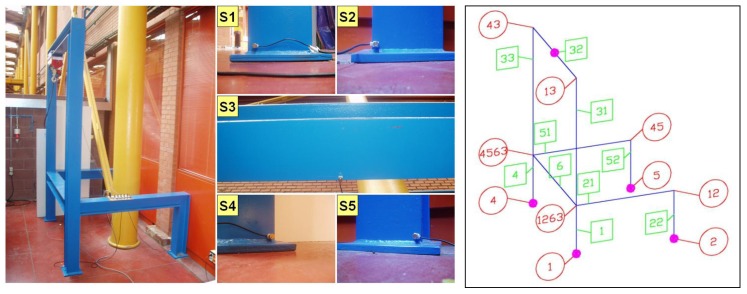
Geometrical description of the structure and sensors.

Finally, the nominal distances between the nodes of the real 3D structure used are shown in Table 1. The non-negligible thickness of bars signifies that it is easy to obtain the minimum and maximum distances between different parts of the structure, by taking into account the dimensions of the normalized profile of each bar (see a general view of the structure used from Figure 7 (left)).

The location of sensors is determined in order to detect delays in the acoustic waves. This location has allowed us to locate impacts on any bar. The optimum number of sensors and their location on a given structure can be determined by using optimization techniques such as functions of precision, duality of solutions, redundancy, and cost. For the 3D structure shown in Figure 7, which is considered as a star with four links with a non-symmetrical central ring, the optimal solution is found with four sensors for each of the tips and an additional sensor for solving dual solutions on the rectangular central loop (ring). However, we have to remark that the optimum number of sensors and their location in a 3D general structure is still unsolved problem.

**Table 1:** Nominal lengths of the experimental structure.

Bar Number	From Node	To Node	Nominal Distance	Steel Profile
1	1	1263	0.695 m	HEB 140
21	1263	12	1.360 m	HEB 130
22	12	2	0.695 m	HEB 130
31	1263	13	1.845 m	HEB 140
32	13	34	1.660 m	HEB 140
33	34	4563	1.845 m	HEB 140
4	4	4563	0.695 m	HEB 140
51	4563	45	1.360 m	HEB 130
52	45	5	0.695 m	HEB 130
6	1263	4563	1.660 m	HEB 160

Figure 7 (right) shows the geometrical model of the structure (blue). In this figure, the bar numbers have been represented inside a green square, and the node numbers have been represented inside a red circle. The location of the sensors is also shown on the right-hand side of Figure 7 as small pink circles. Sensors S_1, S_2, S_4 and S_5 have been located on nodes 1, 2, 4 and 5, respectively. Sensor S_3 ;has been located in the middle of the bar denoted as *32*. Figure 7 (left side) shows the real structure with the sensors and a view of each one too.

## 4. Results

Having briefly described the experimental setup, some experimental results are now presented in this section. Matrix D is obtained from Table 1 and Figure 7. (See Equation (10)):

(16) $$D=\left[\begin{array}{ccccc} 0 & 2.7500 & 3.3700 & 3.0500 & 4.4100\\ 2.7500 & 0 & 4.7300 & 4.4100 & 5.7700\\ 3.3700 & 4.7300 & 0 & 3.3700 & 4.7300\\ 3.0500 & 4.4100 & 3.3700 & 0 & 2.7500\\ 4.4100 & 5.7700 & 4.7300 & 2.7500 & 0 \end{array}\right]\;\;\;\;\;\;\left(m\right)$$

A set of six impacts were produced on the structure. The experimental results of these impacts and the solution used by the proposed system to locate them are presented in the following subsections. Figure 7 (right) shows a topological representation of the structure, the location of the sensors and the approximate position of impact points, also denoted as P_1 to P_6. Points P_1 and P_3 are of special interest owing to the symmetry propagation of the acoustic waves, while points P_4, P_5 and P_6 are studied because of their nearness to node *1263* (see Figure 7 right) and their different treatment.

After obtaining matrix D, a great number of experiments on the structure shown in Figure 7 (left) were carried out in order to calibrate the system and to determine the speed of the sound which has been considered to be between the longitudinal speed and the transversal speed of sound. For all the experiments, presented below, $C_{m}$ has been taken as being:

(17) $$C_{m}=4470.6\;\text{m/s}$$

The achievement of a deterministic, robust, and reliable method for calibrating the direction-dependent wave speed is a challenging issue for anisotropic materials. Even for isotropic materials, depending on the wave modes, the wave speed may be also slightly influenced by structural thickness, stiffening beams or other parameters. Some recent techniques have been proposed to overcome this weak point. (see [27]).

### 4.1. First Impact: Impact on P_1

**Figure 8:**
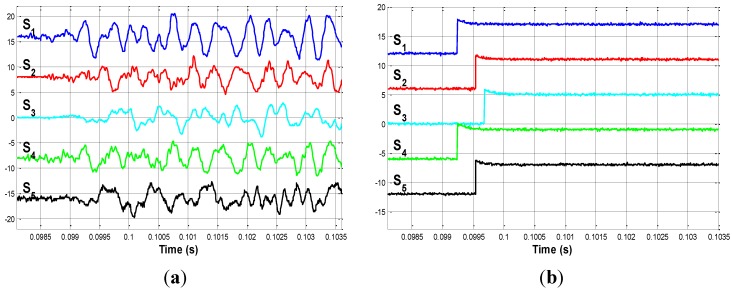
(**a**) Impact on P_1. Detailed view; (**b**) Edge signals when impacting on P_1.

The first impact is produced in the middle of bar 6. This point is called P_1. The signals measured by the sensors are shown in Figure 8a. The delays between the acoustic signals can be clearly appreciated in this figure.

If the instant at which the acoustic waves reach each sensor is observed, the first sensor that detects the signal is S_1. The proposed electronic circuit is responsible for converting these signals into binary edge signals. Figure 8b shows these edge signals; from which the delays from sensor S_1 are easily determined. According to Equation (12), $\Delta t_{1}$ is obtained from these edge signals, and is shown in the following equation:

(18) $$\Delta t_{1}=\left[\begin{array}{ccccc} 0 & 0.2910 & 0.4390 & 0.0050 & 0.2890 \end{array}\right]\cdot 10^{-3}\;\text{s}$$

Sorted incremental time vector $\Delta t_{1\_s}$ is obtained by arranging the elements from $\Delta t_{1}$ in increasing magnitude:

(19) $$\Delta t_{1\_s}=\left[\begin{array}{ccccc} 0 & 0.0050 & 0.2890 & 0.2910 & 0.4390 \end{array}\right]\cdot 10^{-3}\;\text{s}$$

while the correspondence between the elements of $\text{Δ}t_{1\_s}$ and each sensor is:

(20) $$S\_s=\left[\begin{array}{ccccc} 1 & 4 & 5 & 2 & 3 \end{array}\right]$$

The first row of matrix D corresponding to sensor S_1 is denoted as $D_{1}$ in accordance with Equation (13). In this case:

(21) $$D_{1}=\left[\begin{array}{ccccc} 0 & 2.7500 & 3.3700 & 3.0500 & 4.4100 \end{array}\right]\;\text{m}$$

Finally, upon applying Equation (14) and the values from (19), (20) and (21), the following estimated impact positions are obtained (parentheses in the subscripts for the sake of clarity are here avoided):

(22) $$\begin{array}{c} x_{14}=1.5138\;\text{m}\\ x_{15}=1.5590\;\text{m}\\ x_{12}=0.7245\;\text{m}\\ x_{13}=0.7037\;\text{m} \end{array}$$

P_1 is located at 1.525 m following the path between S_1 and S_4 (the distances are obtained from Table 1 and their positions from Figure 7). Equation (19) provides two solutions: $x_{1\left(4\right)}$ and $x_{1\left(5\right)}$, which indicate that the impact point has been located at $x_{1\left(4\right)}=1.5138\;\text{m}$ or $x_{1\left(4\right)}=1.5590\;\text{m}$ with regard to the paths between S_1 and S_4 or between S_1 and S_5, respectively. The solutions provided by $x_{1\left(2\right)}$ and $x_{1\left(3\right)}$ meanwhile indicate that the delays between the paths delimited by S_1 and S_2 or between S_1 and S_3 are equivalent to the delays between the signals from the common node *1263* (see Figure 7 right) to S_1 (which are fixed delays). The symmetry of the structure with regard to P_1 and the symmetry of the delays with regard to S_1, S_2 and S_4, S_5 will be noted.

As the wave speed is very fast, small errors may appear in the identified arrival time causing errors in the identified impact location on the order of 20–30 cm. The proposed system provides the vector of times $\text{Δ}t_{i\_s}$ (see Equation (12)) in ascending order and it is assumed that the measurements are less reliable the greater the value time owing to signal deterioration. Despite this fact, we have to remark that the system achieves redundant measurements (four to be explicit) in the structure in which the impact is produced. Thanks to this measurement redundancy, the measurement errors found can be therefore easily detected and not processed.

### 4.2. Second Impact: Impact on P_2

**Figure 9:**
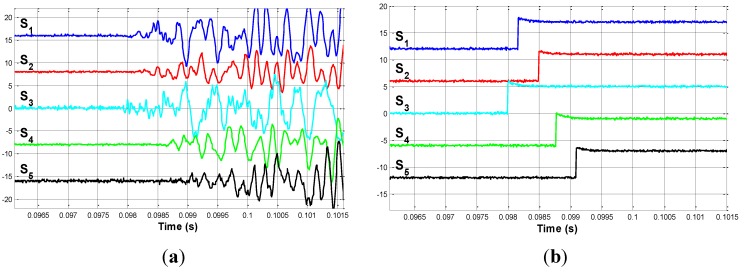
(**a**) Impact on P_2. Detailed view; (**b**) Edge signals when impacting on P_2.

P_2 is located at 2.000 m on the vertical line that joins nodes 1 and 13. The signals provided by sensors are shown in Figure 9a.

In this Figure 9a, it is clear that the first sensor to detect the signal is S_3. Figure 9b shows the edge signals for this second case.

$\Delta t_{3}$ is obtained from these edge signals. Its values are:

(23) $$\Delta t_{3}=\left[\begin{array}{ccccc} 0.1675 & 0.4926 & 0 & 0.7656 & 1.0954 \end{array}\right]\cdot 10^{-3}\;\text{s}$$

After arranging the elements of $\Delta t_{3}$ in increasing magnitude, $\Delta t_{3\_s}$ we obtain:

(24) $$\Delta t_{3\_s}=\left[\begin{array}{ccccc} 0 & 0.1675 & 0.4926 & 0.7656 & 1.0954 \end{array}\right]\cdot 10^{-3}\;\text{s}$$

and the correspondence between the elements of $\Delta t_{3\_s}$ and each sensor is:

(25) $$S\_s=\left[\begin{array}{ccccc} 3 & 1 & 2 & 4 & 5 \end{array}\right]$$

The third row of D corresponding to sensor S_3 after shorting is $D_{3}$:

(26) $$D_{3}=\left[\begin{array}{ccccc} 3.3700 & 4.7300 & 0 & 3.3700 & 4.7300 \end{array}\right]\;\text{m}$$

The estimated impact positions are (Equation (15)):

(27) $$\begin{array}{c} x_{31}=1.3106\;\text{m}\\ x_{32}=1.2639\;\text{m}\\ x_{34}=-0.0264\;\text{m}\\ x_{35}=-0.0836\;\text{m} \end{array}$$

Our system detects the second impact at $x_{3\left(1\right)}=1.3106\;\text{m}$ and $x_{3\left(2\right)}=1.2639\;\text{m}\;$. These distances are measured from S_3 to S_1 or from S_3 to S_2 rather than being measured from S_1. If the S_3 to P_2 location is represented with regard to S_3 rather than S_3 to S_1, this distance becomes S_3 to $P_{2}=1.37\;$instead of $P_{2}=2.00\;\text{m}$. (See Table 1). The errors between solutions $x_{3\left(1\right)}$ and $x_{3\left(2\right)}$ and $P_{2}=1.37\;\text{m}$ are 0.0594 m and 0.1061 m, respectively. These errors are less than or similar to the width of the profiles of the structure (130, 140, and 160 mm), and both solutions are considered to be good solutions.

### 4.3. Third Impact: Impact on P_3

**Figure 10:**
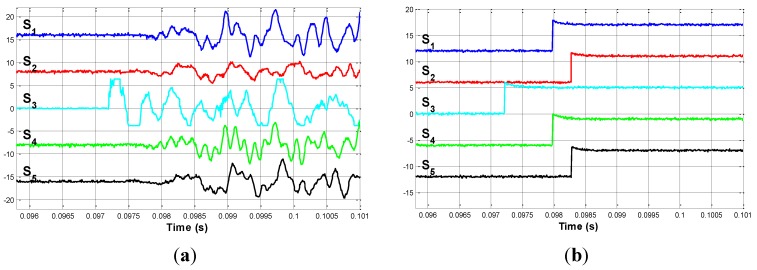
(**a**) Impact on P_3. Detailed view; (**b**) Edge signals when impacting on P_3.

P_3 is located at 0.01 m from S_3. The signals provided by sensors are now shown in Figure 10a. The edge signals for this case are shown in Figure 10b. In this case, the first sensor to detect the impact is of course S_3, and this will be the reference sensor used to measure the delays.

For this third case, $\Delta t_{3}$ and $\Delta t_{3\_s}$ are obtained. Their values are:

(28) $$\Delta t_{3}=\left[\begin{array}{ccccc} 0.7612 & 1.0594 & 0 & 0.7656 & 1.0654 \end{array}\right]\cdot 10^{-3}\;\text{s}$$

(29) $$\Delta t_{3\_s}=\left[\begin{array}{ccccc} 0 & 0.7612 & 0.7656 & 1.0594 & 1.0654 \end{array}\right]\cdot 10^{-3}\;\text{s}$$

$D_{3\_s}$, which corresponds to sensor S_3, is given by Equation (26). The estimated impact positions are (Equation (14)):

(30) $$\begin{array}{c} x_{31}=-0.0165\;\text{m}\\ x_{34}=-0.0264\text{ m}\\ x_{32}=-0.0031\;\text{m}\\ x_{35}=-0.0165\;\text{m} \end{array}$$

The system detects the impact on S_3. In this special case, there is only one set of solutions, all of which indicate the real impact location.

### 4.4. Impact on P_4

**Figure 11:**
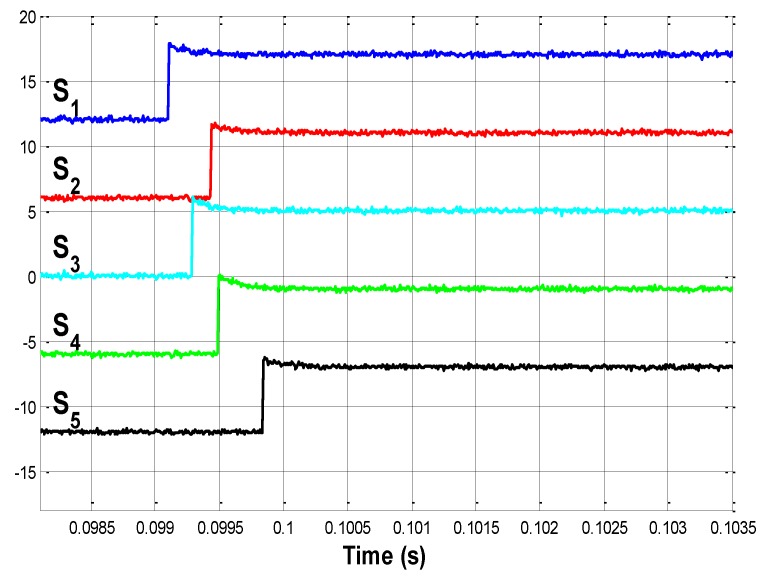
Edge signals when impacting on P_4.

P_4 is located at 1.30 m on the vertical bar that joins nodes 1 and 13. Figure 11 shows the edge signals for this case. Note the similarity between P_4 and P_2 from a geometrical point of view. If the edge signals corresponding to impacts on P_4 and P_2 are analyzed, it is possible to appreciate great differences between them. In this case, the first sensor to detect the impact is S_1 (instead of S_3 when impacting on P_2).

In this fourth case, $\Delta t_{1}$ is:

(31) $$\Delta t_{1}=\left[\begin{array}{ccccc} 0 & 0.3321 & 0.1892 & 0.3993 & 0.7432 \end{array}\right]\cdot 10^{-3}\text{ s}$$

and after again applying again Equation (14) and the values of $D_{1}$ provided by Equation (21), the following solutions are obtained:

(32) $$\begin{array}{c} {\text{x}}_{13}=1.2621\text{ m}\\ {\text{x}}_{12}=0.6327\text{ m}\\ {\text{x}}_{14}=0.6324\text{ m}\\ {\text{x}}_{15}=0.5437\text{ m} \end{array}$$

P_4 is detected along the path between S_1 and S_3. The error between P_4=1.30 m and solution ${\text{x}}_{1\left(3\right)}$ is 0.0379 m. This error is considered to be an acceptable error for the same reasons given in Section 4.2.

### 4.5. Impact on P_5

P_5 is located at 1.20 m from S_1 and follows the path to S_4 through node *1263*. Only $\Delta t_{1}$ values and their solutions are presented in this and the following subsections. $\Delta t_{1}$ is:

(33) $$\Delta t_{1}=\left[\begin{array}{ccccc} 0 & 0.2896 & 0.4413 & 0.1392 & 0.4396 \end{array}\right]\cdot 10^{-3}\;\text{s}$$

The solutions obtained for the impact position detected are:

(34) $$\begin{array}{c} x_{14}=1.2138\;\text{m}\\ x_{12}=0.7277\;\text{m}\\ x_{15}=1.2224\;\text{m}\\ x_{13}=0.6986\;\text{m} \end{array}$$

P_5 is detected along the path between S_1 and S_4 or along the path between S_1 and S_5 (the same path) in accordance with solutions ${\text{x}}_{1\left(4\right)}$ and ${\text{x}}_{1\left(5\right)}$.

### 4.6. Impact on P_6

The last impact point P_6 is located at 1.30 m from S_1 and when following the path to S_2, $\Delta t_{1}$ is:

(35) $$\Delta t_{1}=\left[\begin{array}{ccccc} 0 & 0.0302 & 0.4363 & 0.3540 & 0.6621 \end{array}\right]\cdot 10^{-3}\;\text{s}$$

The solutions obtained (measured from S_1) are:

(36) $$\begin{array}{c} x_{12}=1.3075\;\text{m}\\ x_{14}=0.7337\;\text{m}\\ x_{13}=0.7097\;\text{m}\\ x_{15}=0.7250\;\text{m} \end{array}$$

P_6 is detected along the path between S_1 and S_2. The only solution provided by ${\text{x}}_{1\left(2\right)}$ is considered to be a good solution. The remainder are considered to be fixed delays between node *1263* (see Figure 7 right) and S_1 as in the previous subsections.

## 5. Conclusions

This paper has shown that a proposed low-cost method with which to detect and locate impacts on 3D metal-based structures can easily be integrated into a robotized inspection system and a climbing robot can then inspect the zones at which these collisions have taken place. Based on the time delays of propagation of the acoustic waves along the structure, the proposed system determines the instant at and the position at which the impact has occurred by means of the strategic location of piezoelectric sensors and an electronic-computerized system. The precision and high time response of the proposed system make it very useful for this kind of task. Moreover, the proposed system can easily be implemented in a low cost FPGA system with the analog and opto-isolated stage as a full low-cost system. The combination of this technique and a climbing robot permits the full automation of the task of first detecting, then locating, and finally inspecting the point on a 3D metallic structure on which the collision has occurred. In the case where just the proposed system itself is used (without a robotized inspection system), it allows us to detect and locate the impact on the structure, thus facilitating future human inspection tasks. Experimental results concerning the detection and location of collisions have been presented. The proposed method can be applied to more complex 3D metallic structures, but an optimization process with which to determine the minimum number and optimal location of piezoelectric sensors along a given structure must be solved previously. This will be the topic of our future publications.
